# Advanced Therapy Medicinal Products as Potential Therapeutic Strategy against COVID-19 and Immune-Related Disorders

**DOI:** 10.3390/ijms25053079

**Published:** 2024-03-06

**Authors:** Panagiotis Mallis, Efstathios Michalopoulos, Catherine Stavropoulos-Giokas

**Affiliations:** 1 Hellenic Cord Blood Bank, Biomedical Research Foundation Academy of Athens (BRFAA), 115 27 Athens, Greece; smichal@bioacademy.gr (E.M.); cstavrop@bioacademy.gr (C.S.-G.); 2 Immunology Department & National Tissue Typing Center, General Hospital of Athens “Gennimatas”, 154 Mesogeion Ave., 115 27 Athens, Greece

Advanced Therapy Medicinal Products (ATMPs) comprise a heterogenous class of innovative medicinal products, which further require extensive preclinical and clinical assessments before their broader use in the general population [1]. ATMPs can be either recombinant nucleic acids or engineered cells and tissues [1,2]. Therefore, ATMPs can be classified into four subcategories, including (1) somatic cell therapy medicinal products (SCTMPs), tissue-engineered products (TEPs), gene therapy medicinal products (GTMPs), and combined ATMPs (cATMPs) [3]. The European Union (EU) has a specific legal framework, which, among others, also clearly indicates the differentiation between cell-based products and cell-based therapies. In this context, if human tissues/cells when minimally manipulated, including in vitro processes that do not alter their relevant biological characteristics, such as (a) centrifugation, (b) trimming, (c) flushing, (d) refrigeration, (e) freezing, (f) freeze drying, (g) the use of additives (e.g., cryopreservatives, anticoagulants, antimicrobial agents), (g) or irradiation, are not considered as ATMPs, the active substance cannot be commercialized or manufactured on an industrial scale [3,4]. Therefore, the proper classification of ATMPs defines the regulatory framework further, where the requirements of development and marketing are also included. The EU regulation 1394/2007/EC, in combination with the Directive 2009/120/EU, provides the overall framework and also the updated definitions and detailed scientific/technical requirements of ATMPs [1,2,3,4].

In the context of the authorization and release of ATMPs in the EU, the application of the clinical trial must be prepared and submitted to the national competent authority [3]. Additionally, the Committee for Advanced Therapies (CATs) and the Committee for Medicinal Products for Human Use (CHMP) of the European Medicines Agency (EMA) are responsible for the scientific evaluation and approval of ATMPs. These committees are dedicated to the assurance of the safety, quality, and efficacy of ATMPs, thus performing the draft opinion and the final decision for the release of the ATMPs.

The first released ATMP in the EU was performed in 2009 and constituted a tissue-engineered product known as ChondroCelect^®^, which is intended as a therapeutic treatment of cartilage and bone defects [2]. Accordingly, in the United States (US), PROVENGE^®^, a somatic cell therapy for prostate cancer treatment, was approved as ATMP and released in 2010 [2]. In the field of gene therapy, Glyberia^®^ was the first ATMP of this category that was released in the EU in 2012, which was designed to reverse lipoprotein lipase deficiency (LPLD) in patients suffering from severe pancreatitis.

Providing the main framework where the ATMPs can act beneficially in human disorders, recent applications of the above can also be found in COVID-19 and immune-related diseases. Severe acute respiratory syndrome coronavirus 2 (SARS-CoV-2) is the responsible agent of COVID-19, which was initiated in December 2019 and further escalated into a global pandemic [5]. Based on the World Health Organization (WHO), more than 774 million cases and more than 8.6 million deaths have been reported worldwide. Currently, COVID-19 has spread to more than 220 countries, with the US acting as a leader for new SARS-CoV-2 cases (with more than 103,400,000 confirmed cases) [6]. Moreover, SARS-CoV-2, from its first appearance until today, has undergone a great number of genome mutations, leading to the identification of new variants and subvariants such as Beta, Alpha, and Omicron (BA.2.86, XBB.1.9.1, XBB.2.3, CH.1.1, etc.), accompanied by differences in disease transmission and pathogenesis [7]. Among the different variants, SARS-CoV-2 can induce severe immunopathogenic damage, especially in individuals with immune system defects, elderly populations, or individuals with underlying disorders [7]. It is well known that the infected immune cells, such as macrophages and monocytes by SARS-CoV-2, may initiate the cytokine release syndrome (CRS) [8]. Damaged or infected cells also release damage-associated molecular patterns (DAMPs) and inflammatory cytokines such as interleukin 1β (ΙL-1β), tumor necrosis factor-A (TNF-A), IL-3, and IL-6. This further leads to the overactivation of other immune cells such as dendric cells (DCSs), T helper (Th) 1, Th2, Th17, natural killer (NK) cells and B cells, which produce a great number of inflammatory molecules, e.g., IL-1B, IL-1RA, IL-7, IL-8, IL-9, TNF-A, the fibroblast growth factor (FGF), transforming growth factor-β1 (TGF-β1), granulocyte–macrophage colony-stimulating factor (GM-CSF), IFN-γ, G-CSF, IP10, MCP1, MIP1A, platelet-derived growth factor (PDGF), TNF-α, and vascular endothelial growth factor (VEGF) [8]. In turn, SARS-CoV-2 can spread to the lung parenchyma, infecting, and damaging the alveolar epithelial cells and constituting the primary cause of severe acute respiratory syndrome [8]. The above events lead to a very serious situation, which needs to be properly administrated in order to prevent loss of life.

Moreover, the comprehensive study of SARS-CoV-2 led the scientific society to conclude that common pathogenetic characteristics are also found in other immune-related human disorders, such as autoimmune diseases. Moreover, individual’s genetic background plays a significant role in the underlining resistance against infectious diseases. Specifically, the COVID Human Genetic Effort (http://www.COVIDhge.com, accessed on 25 February 2024) has already reported that 15% of critically ill COVID-19 patients are actively associated with inborn errors of immunity (IEIs) of IFN type I, Toll-like receptor 3 (TLR3) and interferon regulatory factor (IRF) 7 [8]. In addition, more than 10% of critically ill COVID-19 patients were characterized by neutralizing antibodies against interferons, cytokines, and signal-inducing molecules favoring immune responses [8].

Recently, besides the already established protocols, ATMPs have also been considered as candidates for a therapeutic approach, especially for severely ill COVID-19 patients admitted to ICUs. ATMPs can utilize the mesenchymal stromal cells (with known immunoregulatory/immunomodulatory properties) and chimeric antigen receptor (CAR) T and NK cells, for the better administration of COVID-19. To date, the US Food and Drug Administration (FDA) has granted clearance for the performance of phase I/II clinical trials for investigational COVID-19 therapy utilizing allogeneic MSCs [8].

To properly attenuate SARS-CoV-2’s immunopathogenic defects, Gonzalez-Garcia et al. [9] reported the development of CAR-like constructs, which can recognize the ACE2 viral receptor that is responsible for virus entry to the host cells. These CAR-T cells can be obtained after the specific manipulation of patients’ cells and, thus, can be used to prevent the entry of SARS-CoV-2 and also its associated variants despite the genome mutations that it could bear. In the same line, Nova et al. [10] showed the efficient large-scale production of SARS-CoV-2-specific CD4^+^ and CD8^+^ T cells. Specifically, antigenic T-cells prepared by the IFN-γ cytokine capture system (CCS) have been classified as ATMPs, authorized for intended use by the EMA, and are currently used in critically ill SARS-CoV-2 patients. Another study conducted by Piplani et al. [11] and Barakat et al. [12] showed promising evidence regarding the design and synthesis of modern compounds targeting either the ACE2 receptor or the replication machinery of SARS-CoV-2. Moreover, Lee et al. [13] reported the beneficial role of compounds that can downregulate specific signaling pathways, such as the sterile alpha motif, histidine-aspartate domain-containing protein 1 (SAMHD1) tetramerization and the cytosolic DNA sensor cyclic-GMP–AMP synthase (cGAS), as a stimulator of interferon gene (STING) signaling pathways. The above pathways and the involved molecules play crucial roles in SARS-CoV-2 pathogenesis by inducing CRS; therefore, their downregulation may act beneficially in critically ill patients. In the context of developing advanced ATMPs and mediated therapeutic strategies, Khan et al. [14] utilized Cheminformatics to discover potential probe inhibitors of the Omicron subvariant spike protein. The designed probes showed intriguing results in preventing viral entry; therefore, further in vitro and in vivo studies could be performed to more thoroughly assess the efficacy of these compounds against other SARS-CoV-2 subvariants.

In conclusion, ATMPs may represent valuable therapeutic strategies primarily for severely deceased patients. Currently, ATMPs are also applied to immune-related disorders and critically ill COVID-19 patients with beneficial effects. The accomplishment of efforts focused on the production of safe ATMPs may furnish new perspectives on the development of modern therapeutic strategies with a greater immunogenic impact against human disorders, thus providing more options to clinicians.

## Data Availability

Not applicable.
